# Risk prediction models to determine maternal and newborn adverse pregnancy outcomes in low and middle-income countries: A scoping review protocol

**DOI:** 10.1371/journal.pone.0318658

**Published:** 2025-03-24

**Authors:** Douglas Aninng Opoku, Peter Agyei-Baffour, Stephaney Gyaase, Eliezer Odei-Lartey, Joseph Osarfo, Jonathan Gmanyanmi, Francis Appiah, George Adjei, Yeetey Enuameh

**Affiliations:** 1 School of Public Health, Kwame Nkrumah University of Science and Technology, Kumasi, Ghana; 2 Allen Clinic, Family Healthcare Services, Kumasi, Ghana; 3 Kintampo Health Research Centre, Kintampo, Ghana; 4 Department of Community Health, School of Medicine, University of Health and Allied Sciences, Ho, Ghana; 5 Department of Community Medicine, School of Medical Sciences, University of Cape Coast, Cape Coast, Ghana; COMSATS University Islamabad - Lahore Campus, PAKISTAN

## Abstract

**Introduction:**

Globally, low and middle-income countries (LMICs) account for the majority of the adverse pregnancy outcomes. Risk prediction models (RPMs) can guide physicians in making clinical decisions to improve maternal and newborn health. However, there is scanty data on RPMs in determining adverse maternal and newborn outcomes in LMICs. Hence, this scoping review aims to describe the RPMs and the risk factors which have been used to determine both maternal and newborn adverse outcomes of pregnancy in LMICs.

**Methods:**

This scoping review will be guided by the Preferred Reporting Items for Systematic Reviews and Meta-analysis extension for Scoping Reviews (PRISMA-ScR) and the JBI methodology for scoping reviews. The review would employ the Population, Concept, Context (PCC) framework to include studies that reported RPMs to determine either adverse maternal or newborn outcomes of pregnancy or both in LMICs. A literature search will be conducted in four databases for both published and unpublished articles on RPMs for adverse maternal or newborn outcomes from January 1, 2000, to June 26, 2024. We will use the JBI approach for study selection, data extraction, and presentation. The screening and data extraction will be conducted by two independent reviewers.

**Conclusion:**

This scoping review will provide a comprehensive assessment of RPMs for adverse maternal and newborn outcomes in LMICs. This study will help gain knowledge on the up-to-date literature on risk prediction models for adverse pregnancy outcomes which can be useful for researchers and clinicians in making clinical decisions.

**Review registration:** Open Science Framework https://doi.org/10.17605/OSF.IO/B9CKJ.

## Introduction

Risk prediction models (RPMs) are statistical equations or machine learning algorithms that combine several risk factors to predict the occurrence of a health-related outcome in the future [1]. They play a significant role in reducing maternal and newborn morbidity and mortality globally due to their ability to facilitate the timely detection of pregnant women and infants at risk of adverse outcomes [2,3]. Timely prediction of pregnancy complications is crucial for early intervention including referral to a tertiary healthcare centre, early treatments and surveillance, which could help decrease maternal and neonatal morbidity and mortality [4,5]. RPMs can also guide physicians in making informed decisions in the care of pregnant women at risk of adverse pregnancy outcomes (APOs).

Despite progress in reducing APOs such as maternal and neonatal mortality in the last two decades, a World Health Organization’s (WHO) report in 2020 showed a marked-up burden of maternal and neonatal mortalities [6,7]. Globally, about 287,000 and 2.4 million maternal and neonatal (newborn] deaths respectively occurred in 2020 [6,7]. Approximately 95% and 79% of these maternal and neonatal deaths occurred respectively in low and middle-income countries (LMICs) with the burden very severe in sub-Saharan Africa (70% and 43% for maternal and neonatal mortality respectively) [6,7]. Previous studies have also reported a high burden of APOs such as preeclampsia, pregnancy-related haemorrhages, low birth weight and preterm delivery in LMICs [8–11]. There is a need to overcome challenges such as sub-optimal uptake of available effective interventions, poor infrastructure, late booking for antenatal care (ANC), poor ANC attendance and inadequate human resources including a lack of skilled birth attendants and qualified staff to reduce the high burden of maternal and neonatal mortality in LMICs [12–15]. Considering these challenges, a risk prediction model may be useful in the provision of quality maternal and neonatal healthcare to enhance good pregnancy outcomes in LMICs.

The maternal risk factors such as age, obesity, high blood pressure etc. which have previously been reported for classifying the severity of pre-eclampsia by some guidelines in clinical practice could not accurately predict women at risk of developing it in both developing and developed countries [16–18]. A new care model which integrates RPMs may be crucial for reducing the high burden of APOs in LMICs due to its ability to predict an adverse outcome. Despite studies reporting on the risk factors of APOs [19–22], there are still gaps in the methods of predicting the risk of APOs using RPMs, especially in LMICs. The use of traditional methods including logistic and linear regressions for predicting the risk of APOs are unable to handle large datasets with complex interactions and non-linear relationships efficiently [23]. The relationship between several health data may be complex, making a more sophisticated modelling approach like RPMs especially those based on machine learning useful due to its higher predictive power [23]. With the scanty data on RPMs in determining adverse maternal and newborn outcomes in LMICs, this review will provide timely evidence from a scoping review of RPMs used in determining APOs. On 5th January 2024, a preliminary search conducted in MEDLINE, PROSPERO, JBI Evidence Synthesis and Cochrane Database of Systematic Reviews did not find any existing or in-progress scoping or systematic reviews on the topic.

This scoping review aims to describe the RPMs and the risk factors which have been used to determine both maternal and newborn adverse outcomes of pregnancy in LMICs. This review will provide significant data which can enhance clinical and scientific development and improve birth and maternal health outcomes in LMICs.

## Review questions

The review will be guided by the following research questions:

- What RPMs have been used to predict APOs in LMICs?
- What factors have been used in RPMs to predict APOs in LMICs?
- What are the research gaps in factors related to APO predictions in LMICs?

### Inclusion criteria

The review would employ the Population, Concept, Context [PCC] framework

### Population

This review will consider all studies that include APOs. This will include studies that reported on adverse maternal or newborn outcomes or both. Adverse maternal outcomes in this review will be defined as undesirable health issues for the mother during pregnancy, labour, delivery and postpartum periods. On the other hand, adverse newborn outcomes in this study will be defined as unwanted health problems for the newborn during pregnancy, labour, delivery and postpartum periods. The studies will be eligible for inclusion irrespective of the age, gravidity and parity of pregnant women.

### Concept

This review will consider studies that reported risk factors for APOs including demographic characteristics, social, ecological, clinical, behavioural etc. in RPMs. The eligibility of studies in this review will be based on satisfying two criteria: first, should report on the risk factors of APOs and two, these risk factors should be used in RPMs to predict APOs. Other terminologies for APOs will include adverse birth outcomes, poor birth outcomes, poor neonatal outcomes, poor maternal outcomes, poor obstetric outcomes, adverse obstetric outcomes, adverse maternal outcomes, adverse newborn outcomes and adverse neonatal outcomes.

### Context

This review will consider studies conducted in LMICs based on the World Bank’s classification [24].

### Types of sources

This review will consider all quantitative studies that reported RPMs to determine either adverse maternal or newborn outcomes or both in LMICs as defined by the World Bank [24] between 1st January 2000 and 26th June 2024. The selection of this timeline was based on the preference of the authors. The studies should have been published in either peer-reviewed journals or grey literature and in English language. APOs that will be considered for this review will be studies that either reported any adverse maternal or newborn outcomes or a composite variable.

All protocols, abstracts, conference papers, commentaries and unavailable full texts will be excluded from this review.

## Methods

This scoping review will be guided by the Preferred Reporting Items for Systematic Reviews and Meta-analysis extension for Scoping Reviews (PRISMA-ScR) (see S1 File) and the JBI methodology for scoping reviews [25,26].

### Search strategy

The search strategy will aim to retrieve both published and unpublished articles. The following databases will be searched: PubMed/Medline, Cochran Library, Web of Science and Scopus. Grey literature will also be searched.

This review will adopt a three-stage search strategy based on JBI guidelines [27]: phase one (initial search), phase two (second search using identified keywords) and phase three (third search which includes a review of references from identified articles for critical appraisal). In phase one, an initial search of databases was conducted to find articles on the topic. A comprehensive search technique for MEDLINE (PubMed) was created using the text words found in titles and abstracts as well as index keywords of relevant studies (Table 1). This will be followed up with an analysis of text words in the title, abstract and index terms adopted for the article. This will enhance the development of a search strategy that will suit each database source. In the second phase, the identified keywords and index terms will be used to search for literature in all relevant databases. In the third phase, a comprehensive search will be conducted by reviewing the reference list of all the identified articles for critical appraisal for additional eligible studies (snowballing).

**Table 1:** Search strategy. MEDLINE (PubMed). Search conducted: June 26, 2024.

Search	Query	Records retrieved
#1	((Angola) OR (Algeria) OR (Bangladesh) OR (Benin) OR (Bhutan) OR (Bolivia) OR (Cabo Verde) OR (Cambodia) OR (Cameroon) OR (Comoros) OR (Congo, Republic) OR (Djibouti) OR (Côte d’Ivoire) OR (Egypt, Arab Republic) OR (Eswatini) OR (Ghana) OR (Guinea) OR (Haiti) OR (Honduras) OR (Jordan) OR (India) OR (Iran, Islamic Republic) OR (Kenya) OR (Kiribati) OR (Kyrgyz Republic) OR (Lao PDR) OR (Lebanon) OR (Lesotho) OR (Micronesia, Fed. Sts.) OR (Mongolia) OR (Morocco) OR (Nepal) OR (Myanmar) OR (Nicaragua) OR (Nigeria) OR (Pakistan) OR (Papua New Guinea) OR (Philippines) OR (Tajikistan) OR (Timor-Leste) OR (Tunisia) OR (Ukraine) OR (Uzbekistan) OR (Vanuatu) OR (Vietnam) OR (Zambia) OR (Zimbabwe)) AND ((((((machine learning) OR (deep learning)) OR (artificial intelligence)) OR (risk prediction)) OR (risk prediction model)) AND ((((((((((((adverse pregnancy outcomes prediction) OR (adverse obstetric outcomes prediction)) OR (poor obstetric outcomes prediction)) OR (poor birth outcomes prediction)) OR (adverse birth outcomes prediction)) OR (adverse maternal outcomes prediction)) OR (adverse neonatal outcomes)) OR (adverse newborn outcomes prediction)) OR (poor neonatal outcomes prediction)) OR (poor newborn outcomes prediction)) OR (poor maternal outcomes prediction)) OR (pregnancy-related complications prediction))) Filters: from 2000/1/1 - 2024/6/26	893
#2	((“angola”[MeSH Terms] OR “angola”[All Fields] OR “angola s”[All Fields] OR (“algeria”[MeSH Terms] OR “algeria”[All Fields]) OR (“bangladesh”[MeSH Terms] OR “bangladesh”[All Fields] OR “bangladesh s”[All Fields]) OR (“benin”[MeSH Terms] OR “benin”[All Fields] OR “benin s”[All Fields]) OR (“bhutan”[MeSH Terms] OR “bhutan”[All Fields] OR “bhutan s”[All Fields]) OR (“bolivia”[MeSH Terms] OR “bolivia”[All Fields]) OR (“cabo verde”[MeSH Terms] OR (“cabo”[All Fields] AND “verde”[All Fields]) OR “cabo verde”[All Fields]) OR (“cambodia”[MeSH Terms] OR “cambodia”[All Fields] OR “cambodia s”[All Fields]) OR (“cameroon”[MeSH Terms] OR “cameroon”[All Fields] OR “cameroons”[All Fields] OR “cameroon s”[All Fields]) OR (“comoros”[MeSH Terms] OR “comoros”[All Fields] OR “comoro”[All Fields]) OR ((“congo”[MeSH Terms] OR “congo”[All Fields]) AND (“republic”[All Fields] OR “republic s”[All Fields] OR “republics”[All Fields])) OR (“djibouti”[MeSH Terms] OR “djibouti”[All Fields]) OR (“cote d ivoire”[MeSH Terms] OR (“cote”[All Fields] AND “d ivoire”[All Fields]) OR “cote d ivoire”[All Fields]) OR ((“egypt”[MeSH Terms] OR “egypt”[All Fields] OR “egypt s”[All Fields]) AND (“arabs”[MeSH Terms] OR “arabs”[All Fields] OR “arab”[All Fields]) AND (“republic”[All Fields] OR “republic s”[All Fields]	893
	OR “republics”[All Fields])) OR (“eswatini”[MeSH Terms] OR “eswatini”[All Fields]) OR (“ghana”[MeSH Terms] OR “ghana”[All Fields] OR “ghana s”[All Fields]) OR (“guinea”[MeSH Terms] OR “guinea”[All Fields] OR “guinea s”[All Fields] OR “guineas”[All Fields]) OR (“haiti”[MeSH Terms] OR “haiti”[All Fields] OR “haiti s”[All Fields]) OR (“honduras”[MeSH Terms] OR “honduras”[All Fields]) OR (“jordan”[MeSH Terms] OR “jordan”[All Fields]) OR (“india”[MeSH Terms] OR “india”[All Fields] OR “india s”[All Fields] OR “indias”[All Fields]) OR ((“iran”[MeSH Terms] OR “iran”[All Fields]) AND (“islam”[MeSH Terms] OR “islam”[All Fields] OR “islamic”[All Fields] OR “islam s”[All Fields] OR “islamism”[All Fields]) AND (“republic”[All Fields] OR “republic s”[All Fields] OR “republics”[All Fields])) OR (“kenya”[MeSH Terms] OR “kenya”[All Fields] OR “kenya s”[All Fields]) OR (“micronesia”[MeSH Terms] OR “micronesia”[All Fields] OR “kiribati”[All Fields]) OR (“kyrgyzstan”[MeSH Terms] OR “kyrgyzstan”[All Fields] OR (“kyrgyz”[All Fields] AND “republic”[All Fields]) OR “kyrgyz republic”[All Fields]) OR (“Lao”[All Fields] AND “PDR”[All Fields]) OR (“lebanon”[MeSH Terms] OR “lebanon”[All Fields] OR “lebanon s”[All Fields]) OR (“lesotho”[MeSH Terms] OR “lesotho”[All Fields] OR “lesotho s”[All Fields]) OR ((“micronesia”[MeSH Terms] OR “micronesia”[All Fields]) AND “fed”[All Fields]	
	AND “sts”[All Fields]) OR (“mongolia”[MeSH Terms] OR “mongolia”[All Fields] OR “mongolia s”[All Fields]) OR (“morocco”[MeSH Terms] OR “morocco”[All Fields]) OR (“nepal”[MeSH Terms] OR “nepal”[All Fields] OR “nepal s”[All Fields]) OR (“myanmar”[MeSH Terms] OR “myanmar”[All Fields] OR “myanmar s”[All Fields] OR “myanmars”[All Fields]) OR (“nicaragua”[MeSH Terms] OR “nicaragua”[All Fields] OR “nicaragua s”[All Fields]) OR (“nigeria”[MeSH Terms] OR “nigeria”[All Fields] OR “nigeria s”[All Fields]) OR (“pakistan”[MeSH Terms] OR “pakistan”[All Fields] OR “pakistan s”[All Fields]) OR (“papua new guinea”[MeSH Terms] OR (“papua”[All Fields] AND “new”[All Fields] AND “guinea”[All Fields]) OR “papua new guinea”[All Fields]) OR (“philippine”[All Fields] OR “philippines”[MeSH Terms] OR “philippines”[All Fields]) OR (“tajikistan”[MeSH Terms] OR “tajikistan”[All Fields]) OR (“timor leste”[MeSH Terms] OR “timor leste”[All Fields] OR (“timor”[All Fields] AND “leste”[All Fields]) OR “timor leste”[All Fields]) OR (“tunisia”[MeSH Terms] OR “tunisia”[All Fields]) OR (“ukraine”[MeSH Terms] OR “ukraine”[All Fields] OR “ukraine s”[All Fields]) OR (“uzbekistan”[MeSH Terms] OR “uzbekistan”[All Fields]) OR (“vanuatu”[MeSH Terms] OR “vanuatu”[All Fields]) OR (“vietnam”[MeSH Terms] OR “vietnam”[All Fields] OR “vietnam s”[All Fields]) OR (“zambia”[MeSH Terms] OR “zambia”[All Fields] OR “zambia s”[All Fields]) OR (“zimbabwe”[MeSH Terms] OR “zimbabwe”[All Fields] OR “zimbabwe s”[All Fields])) AND ((“machine learning”[MeSH Terms] OR (“machine”[All Fields] AND “learning”[All Fields]) OR “machine learning”[All Fields] OR (“deep learning”[MeSH Terms] OR (“deep”[All Fields] AND “learning”[All Fields]) OR “deep learning”[All Fields]) OR (“artificial intelligence”[MeSH Terms] OR (“artificial”[All Fields] AND “intelligence”[All Fields]) OR “artificial intelligence”[All Fields]) OR ((“risk”[MeSH Terms] OR “risk”[All Fields]) AND (“predict”[All Fields] OR “predictabilities”[All Fields] OR “predictability”[All Fields] OR “predictable”[All Fields] OR “predictably”[All Fields] OR “predicted”[All Fields] OR “predicting”[All Fields] OR “prediction”[All Fields] OR “predictions”[All Fields] OR “predictive”[All Fields] OR “predictively”[All Fields] OR “predictiveness”[All Fields] OR “predictives”[All Fields] OR “predictivities”[All Fields] OR “predictivity”[All Fields] OR “predicts”[All Fields]))	
	OR ((“risk”[MeSH Terms] OR “risk”[All Fields]) AND (“predict”[All Fields] OR “predictabilities”[All Fields] OR “predictability”[All Fields] OR “predictable”[All Fields] OR “predictably”[All Fields] OR “predicted”[All Fields] OR “predicting”[All Fields] OR “prediction”[All Fields] OR “predictions”[All Fields] OR “predictive”[All Fields] OR “predictively”[All Fields] OR “predictiveness”[All Fields] OR “predictives”[All Fields] OR “predictivities”[All Fields] OR “predictivity”[All Fields] OR “predicts”[All Fields]) AND (“model”[All Fields] OR “model s”[All Fields] OR “modeled”[All Fields] OR “modeler”[All Fields] OR “modeler s”[All Fields] OR “modelers”[All Fields] OR “modeling”[All Fields] OR “modelings”[All Fields] OR “modelization”[All Fields] OR “modelizations”[All Fields] OR “modelize”[All Fields] OR “modelized”[All Fields] OR “modelled”[All Fields] OR “modeller”[All Fields] OR “modellers”[All Fields] OR “modelling”[All Fields] OR “modellings”[All Fields] OR “models”[All Fields]))) AND (((“adverse”[All Fields] OR “adversely”[All Fields] OR “adverses”[All Fields]) AND (“pregnancy outcome”[MeSH Terms] OR (“pregnancy”[All Fields] AND “outcome”[All Fields]) OR “pregnancy outcome”[All Fields]	
	OR (“pregnancy”[All Fields] AND “outcomes”[All Fields]) OR “pregnancy outcomes”[All Fields]) AND (“predict”[All Fields] OR “predictabilities”[All Fields] OR “predictability”[All Fields] OR “predictable”[All Fields] OR “predictably”[All Fields] OR “predicted”[All Fields] OR “predicting”[All Fields] OR “prediction”[All Fields] OR “predictions”[All Fields] OR “predictive”[All Fields] OR “predictively”[All Fields] OR “predictiveness”[All Fields] OR “predictives”[All Fields] OR “predictivities”[All Fields] OR “predictivity”[All Fields] OR “predicts”[All Fields])) OR ((“adverse”[All Fields] OR “adversely”[All Fields] OR “adverses”[All Fields]) AND (“obstetric”[All Fields] OR “obstetrically”[All Fields] OR “obstetrics”[MeSH Terms] OR “obstetrics”[All Fields] OR “obstetrical”[All Fields]) AND (“outcome”[All Fields] OR “outcomes”[All Fields]) AND (“predict”[All Fields] OR “predictabilities”[All Fields] OR “predictability”[All Fields] OR “predictable”[All Fields] OR “predictably”[All Fields] OR “predicted”[All Fields] OR “predicting”[All Fields] OR “prediction”[All Fields] OR “predictions”[All Fields] OR “predictive”[All Fields] OR “predictively”[All Fields] OR “predictiveness”[All Fields] OR “predictives”[All Fields] OR “predictivities”[All Fields]	
	OR “predictivity”[All Fields] OR “predicts”[All Fields])) OR ((“poverty”[MeSH Terms] OR “poverty”[All Fields] OR “poor”[All Fields]) AND (“obstetric”[All Fields] OR “obstetrically”[All Fields] OR “obstetrics”[MeSH Terms] OR “obstetrics”[All Fields] OR “obstetrical”[All Fields]) AND (“outcome”[All Fields] OR “outcomes”[All Fields]) AND (“predict”[All Fields] OR “predictabilities”[All Fields] OR “predictability”[All Fields] OR “predictable”[All Fields] OR “predictably”[All Fields] OR “predicted”[All Fields] OR “predicting”[All Fields] OR “prediction”[All Fields] OR “predictions”[All Fields] OR “predictive”[All Fields] OR “predictively”[All Fields] OR “predictiveness”[All Fields] OR “predictives”[All Fields] OR “predictivities”[All Fields] OR “predictivity”[All Fields] OR “predicts”[All Fields])) OR ((“poverty”[MeSH Terms] OR “poverty”[All Fields] OR “poor”[All Fields]) AND (“birth s”[All Fields] OR “birthed”[All Fields] OR “birthing”[All Fields] OR “parturition”[MeSH Terms] OR “parturition”[All Fields] OR “birth”[All Fields] OR “births”[All Fields]) AND (“outcome”[All Fields] OR “outcomes”[All Fields]) AND (“predict”[All Fields] OR “predictabilities”[All Fields] OR “predictability”[All Fields] OR “predictable”[All Fields] OR “predictably”[All Fields] OR “predicted”[All Fields] OR “predicting”[All Fields] OR “prediction”[All Fields] OR “predictions”[All Fields] OR “predictive”[All Fields] OR “predictively”[All Fields] OR “predictiveness”[All Fields] OR “predictives”[All Fields] OR “predictivities”[All Fields] OR “predictivity”[All Fields] OR “predicts”[All Fields])) OR ((“pregnancy complications”[MeSH Terms] OR (“pregnancy”[All Fields] AND “complications”[All Fields]) OR “pregnancy complications”[All Fields] OR (“adverse”[All Fields] AND “birth”[All Fields] AND “outcomes”[All Fields]) OR “adverse birth outcomes”[All Fields]) AND (“predict”[All Fields] OR “predictabilities”[All Fields] OR “predictability”[All Fields] OR “predictable”[All Fields] OR “predictably”[All Fields] OR “predicted”[All Fields] OR “predicting”[All Fields] OR “prediction”[All Fields] OR “predictions”[All Fields] OR “predictive”[All Fields] OR “predictively”[All Fields] OR “predictiveness”[All Fields] OR “predictives”[All Fields] OR “predictivities”[All Fields] OR “predictivity”[All Fields] OR “predicts”[All Fields])) OR ((“adverse”[All Fields] OR “adversely”[All Fields] OR “adverses”[All Fields]) AND (“maternally”[All Fields] OR “maternities”[All Fields] OR “maternity”[All Fields] OR “mothers”[MeSH Terms] OR “mothers”[All Fields] OR “maternal”[All Fields]) AND (“outcome”[All Fields] OR “outcomes”[All Fields]) AND (“predict”[All Fields] OR “predictabilities”[All Fields] OR “predictability”[All Fields] OR “predictable”[All Fields] OR “predictably”[All Fields] OR “predicted”[All Fields] OR “predicting”[All Fields] OR “prediction”[All Fields] OR “predictions”[All Fields] OR “predictive”[All Fields] OR “predictively”[All Fields] OR “predictiveness”[All Fields] OR “predictives”[All Fields] OR “predictivities”[All Fields] OR “predictivity”[All Fields] OR “predicts”[All Fields])) OR ((“adverse”[All Fields] OR “adversely”[All Fields] OR “adverses”[All Fields]) AND (“infant, newborn”[MeSH Terms] OR (“infant”[All Fields] AND “newborn”[All Fields]) OR “newborn infant”[All Fields] OR “neonatal”[All Fields] OR “neonate”[All Fields] OR “neonates”[All Fields] OR “neonatality”[All Fields] OR “neonatals”[All Fields] OR “neonate s”[All Fields]) AND (“outcome”[All Fields] OR “outcomes”[All Fields])) OR ((“adverse”[All Fields] OR “adversely”[All Fields] OR “adverses”[All Fields]) AND (“infant, newborn”[MeSH Terms] OR (“infant”[All Fields] AND “newborn”[All Fields]) OR “newborn infant”[All Fields] OR “newborn”[All Fields] OR “newborns”[All Fields] OR “newborn s”[All Fields]) AND (“outcome”[All Fields] OR “outcomes”[All Fields]) AND (“predict”[All Fields] OR “predictabilities”[All Fields] OR “predictability”[All Fields] OR “predictable”[All Fields] OR “predictably”[All Fields] OR “predicted”[All Fields] OR “predicting”[All Fields] OR “prediction”[All Fields] OR “predictions”[All Fields] OR “predictive”[All Fields] OR “predictively”[All Fields] OR “predictiveness”[All Fields] OR “predictives”[All Fields] OR “predictivities”[All Fields] OR “predictivity”[All Fields] OR “predicts”[All Fields])) OR ((“poverty”[MeSH Terms] OR “poverty”[All Fields] OR “poor”[All Fields]) AND (“infant, newborn”[MeSH Terms]	
	OR (“infant”[All Fields] AND “newborn”[All Fields]) OR “newborn infant”[All Fields] OR “neonatal”[All Fields] OR “neonate”[All Fields] OR “neonates”[All Fields] OR “neonatality”[All Fields] OR “neonatals”[All Fields] OR “neonate s”[All Fields]) AND (“outcome”[All Fields] OR “outcomes”[All Fields]) AND (“predict”[All Fields] OR “predictabilities”[All Fields] OR “predictability”[All Fields] OR “predictable”[All Fields] OR “predictably”[All Fields] OR “predicted”[All Fields] OR “predicting”[All Fields] OR “prediction”[All Fields] OR “predictions”[All Fields] OR “predictive”[All Fields] OR “predictively”[All Fields] OR “predictiveness”[All Fields] OR “predictives”[All Fields] OR “predictivities”[All Fields] OR “predictivity”[All Fields] OR “predicts”[All Fields])) OR ((“poverty”[MeSH Terms] OR “poverty”[All Fields] OR “poor”[All Fields]) AND (“infant, newborn”[MeSH Terms] OR (“infant”[All Fields] AND “newborn”[All Fields]) OR “newborn infant”[All Fields] OR “newborn”[All Fields] OR “newborns”[All Fields] OR “newborn s”[All Fields]) AND (“outcome”[All Fields] OR “outcomes”[All Fields]) AND (“predict”[All Fields] OR “predictabilities”[All Fields] OR “predictability”[All Fields] OR “predictable”[All Fields] OR “predictably”[All Fields] OR “predicted”[All Fields] OR “predicting”[All Fields] OR “prediction”[All Fields] OR “predictions”[All Fields] OR “predictive”[All Fields] OR “predictively”[All Fields] OR “predictiveness”[All Fields] OR “predictives”[All Fields] OR “predictivities”[All Fields] OR “predictivity”[All Fields] OR “predicts”[All Fields])) OR ((“poverty”[MeSH Terms] OR “poverty”[All Fields] OR “poor”[All Fields]) AND (“maternally”[All Fields] OR “maternities”[All Fields] OR “maternity”[All Fields] OR “mothers”[MeSH Terms] OR “mothers”[All Fields]	
	OR “maternal”[All Fields]) AND (“outcome”[All Fields] OR “outcomes”[All Fields]) AND (“predict”[All Fields] OR “predictabilities”[All Fields] OR “predictability”[All Fields] OR “predictable”[All Fields] OR “predictably”[All Fields] OR “predicted”[All Fields] OR “predicting”[All Fields] OR “prediction”[All Fields] OR “predictions”[All Fields] OR “predictive”[All Fields] OR “predictively”[All Fields] OR “predictiveness”[All Fields] OR “predictives”[All Fields] OR “predictivities”[All Fields] OR “predictivity”[All Fields] OR “predicts”[All Fields])) OR (“pregnancy-related”[All Fields] AND (“complicances”[All Fields] OR “complicate”[All Fields] OR “complicated”[All Fields] OR “complicates”[All Fields] OR “complicating”[All Fields] OR “complication”[All Fields] OR “complication s”[All Fields] OR “complications”[MeSH Subheading] OR “complications”[All Fields]) AND (“predict”[All Fields] OR “predictabilities”[All Fields] OR “predictability”[All Fields] OR “predictable”[All Fields] OR “predictably”[All Fields] OR “predicted”[All Fields] OR “predicting”[All Fields] OR “prediction”[All Fields] OR “predictions”[All Fields] OR “predictive”[All Fields] OR “predictively”[All Fields] OR “predictiveness”[All Fields] OR “predictives”[All Fields] OR “predictivities”[All Fields] OR “predictivity”[All Fields] OR “predicts”[All Fields]))))) AND (2000/1/1:2024/6/26[pdat])	

### Study selection

The study selection will be guided by the inclusion and exclusion criteria (see Table 2). The criteria may be adjusted by the reviewers where necessary and reported in the final review. After the search, all identified studies will be collated and uploaded into Rayyan Software (http://rayyan.qcri.org) and all records of duplicates will be removed. The title and abstract of the studies screening will be done by two independent reviewers based on the inclusion criteria for the review as well as addressing the research questions. All potentially eligible studies which will be identified including those without a full text will be retrieved and their details will be imported into the JBI SUMARI software [26]. The independent reviewers will retrieve the full text of all the selected studies and evaluate them based on the inclusion criteria. All studies that do not meet the inclusion criteria for the review will be excluded and reasons given as an appendix in the final scoping review report. All the studies that will be included will be critically reviewed independently by two reviewers and in instances where there is a disagreement between the two reviewers for the inclusion of a study, discussions will be made until a consensus is reached, and where necessary assessment of the full text will be done. If there is no agreement between the two reviewers, a third reviewer (senior colleague) will be invited for resolution. The PRISMA flow diagram [28] will be used for the presentation of the results of the search in the final scoping review.

**Table 2:** Inclusion and exclusion criteria.

	Inclusion	Exclusion
Year of publication	January 2000 and 26th June 2024	
Language	English	Other languages
Population	APOs (maternal and newborns)	N/A
Concept	Studies that reported risk factors of APOs using an RPM	N/A
Context	LMICs	Regions outside LMICs, mixed settings where the results were not stratified according to the setting or region
Study type	Quantitative study	

### Data extraction

Data extraction will be conducted by two independent reviewers using a standardized JBI SUMARI extraction tool. The tool will be used to train the research team members before it is used in the final work. The data that will be extracted will include the population (characteristics or predictors such as pregnant women, age, etc.) concept (risk prediction models), context (LMICs), study methods and designs, and other variables relevant to the review questions and objectives. The data extracted by the two independent reviewers will be compared and where there will be disagreements, it will be resolved through a discussion or a third reviewer will be invited to resolve it. In situations where there is missing data in a primary study, the corresponding or a co-author will be contacted by email. However, the available data in the primary study will be used in instances where the corresponding or co-author does not respond to the email and acknowledge the missing data as a study limitation.

### Data analysis and presentation

The extracted data from the studies will be presented using tables based on the objectives and the review questions. An initial summary of the characteristics of the studies including the study design, date of publication, population and authors’ geographical location will be described in the review. Quantitative data will be described descriptively and presented using frequencies. Tables will be used to describe adverse maternal and newborn outcomes, RPMs used to predict these adverse outcomes as well as the predictors that were used for the prediction. We will merge data from the same studies.

A narrative summary will be used to describe the results based on the research questions that guided the review which can guide future development, reporting and comparison of models and outcome selection of adverse maternal and newborn outcomes. We will publish the outcome of this scoping review in a peer-reviewed journal as well as present at either national or international conferences where possible.

## Discussion

This scoping review protocol aims at mapping the existing knowledge on RPMs for adverse maternal and newborn outcomes in LMICs. LMICs continue to dominate the global burden of APO such as low birth weight, preterm delivery, and neonatal and maternal mortalities despite advancements in health technology [6,7]. The ability to predict potential complications ahead of time and provide timely intervention could be useful for reducing pregnancy-related risks. Hence, the application of RPMs is necessary for ensuring the health and safety of both mothers and infants globally.

This review will map out all the available evidence on RPMs to enhance knowledge to improve birth outcomes in LMICs and beyond. The outcomes of this review will be discussed by comparing it to earlier studies. Again, the discussion will be done in connection to potential strategies, research and healthcare delivery aimed at improving the health and safety of mothers and newborns.

A potential limitation of this scoping review will be not accessing the methodological quality of the primary studies that will be included. This is in line with standard practice or guidelines as scoping reviews do not focus on evaluating the methodological quality of included studies [29]. The inclusion of studies with different study designs and methodologies could affect the synthesis of the results due to potential variability among them. Despite these limitations, this review will provide up-to-date literature and knowledge gaps on RPMs for APOs in LMICs. This scoping review will also provide evidence-based data to aid in the development of potential strategies to enhance the health and safety of mothers and newborns.

## Supporting information

S1 File PRISMA-SCR checklist. (DOCX)
