# Supplementary material for: Risk prediction models to determine maternal and newborn adverse pregnancy outcomes in low and middle-income countries: A scoping review protocol
Source: PLoS One. 2025 Mar 24;20(3):e0318658. doi: 10.1371/journal.pone.0318658 (PMC11932470; doi:10.1371/journal.pone.0318658)
Supplement: S1 File — (DOCX) [file pone.0318658.s001.docx]

**PRISMA-P (Preferred Reporting Items for Systematic review and Meta-Analysis Protocols) 2015 checklist: recommended items to address in a systematic review protocol***

| Section and topic | Item No | Checklist item |
| --- | --- | --- |
| ADMINISTRATIVE INFORMATION | | |
| Title: |  |  |
| Identification | 1a | Risk prediction models to determine maternal and newborn adverse pregnancy outcomes in low and middle-income countries: A scoping review protocol |
| Update | 1b |  |
| Registration | 2 | This scoping review protocol has been developed and submitted to Open Science Framework with a DOI of <https://doi.org/10.17605/OSF.IO/B9CKJ> |
| Authors: |  |  |
| Contact | 3a | Douglas Aninng Opoku*^,1,2^, Peter Agyei-Baffour^1^, Stephaney Gyaase^3^, Eliezer Odei-Lartey^3^, Joseph Osarfo^4^, Jonathan Gmanyanmi^1^, Francis Appiah^1^, George Adjei^5^, & Yeetey Enuameh^1^  **Affiliations;**  ^1^School of Public Health, Kwame Nkrumah University of Science and Technology, Kumasi, Ghana  ^2^Allen Clinic, Family Healthcare Services, Kumasi, Ghana  ^3^Kintampo Health Research Centre, Kintampo, Ghana  ^4^Department of Community Health, School of Medicine, University of Health and Allied Sciences, Ho, Ghana  ^5^Department of Community Medicine, School of Medical Sciences, University of Cape Coast, Cape Coast, Ghana |
| Contributions | 3b | Conceptualization: DAO & YE  Data curation: DAO, PA-B, SG, JG & EO-L  Formal analysis: DAO, SG, JO, EO-L & YE  Investigation: DAO, FA, JG & GA  Methodology: DAO, PA-B, SG, JG, JO, FA, GA, EO-L & YE  Supervision: PA-B, GA & YE  Validation: DAO, & JG  Writing – original draft: DAO, FA, JO, & YE  Writing – review and editing: DAO, PA-B, SG, JG, JO, FA, GA, EO-L & YE |
| Amendments | 4 | Not applicable |
| Support: |  |  |
| Sources | 5a | The review is supported by Bill and Melinda Gates Foundation grant number INV-002220" through the Kintampo Health Research Centre, AdOPT Africa Study Training Scholarship. |
| Sponsor | 5b | Kintampo Health Research Centre, AdOPT Africa Study Training Scholarship |
| Role of sponsor or funder | 5c | The funders were not involved in the conceptualization of this scoping review protocol, and they will not be involved in carrying out the review itself. |
| INTRODUCTION | | |
| Rationale | 6 | The maternal risk factors which have previously been reported for classifying the severity of pregnancy complications by some guidelines in clinical practice could not accurately predict women at risk of developing APOs in both developing and developed countries (16–18) A new care model which integrates RPMs may be crucial for reducing the high burden of APOs in LMICs due to its ability to predict an adverse outcome. Despite studies reporting on the risk factors of APOs (19–22), there are still gaps in the methods of predicting the risk of APOs using RPMs, especially in LMICs. With the scanty data on RPMs in determining adverse maternal and newborn outcomes in LMICs, this review will provide timely evidence from a scoping review of RPMs used in determining APOs |
| Objectives | 7 | This scoping review aims to describe the RPMs and the risk factors which have been used to determine both maternal and newborn adverse outcomes of pregnancy in LMICs. This review will provide significant data which can enhance clinical and scientific development and improve birth and maternal health outcomes in LMICs.    **Review questions**  The review will be guided by the following research questions:   1. What RPMs have been used to predict APOs in LMICs? 2. What factors have been used in RPMs to predict APOs in LMICs?   What are the research gaps in factors related to APO predictions in LMICs? |
| METHODS | | |
| Eligibility criteria | 8 | **Inclusion criteria**  The review would employ the Population, Concept, Context (PCC) framework.  **Population**  This review will consider all studies that include adverse birth outcomes. This will include studies that reported on adverse maternal or newborn outcomes or both. Adverse maternal outcomes in this review will be defined as undesirable health issues for the mother during pregnancy, labour, delivery and postpartum periods. On the other hand, adverse newborn outcomes in this study will be defined as unwanted health problems for the newborn during pregnancy, labour, delivery and postpartum periods. The studies will be eligible for inclusion irrespective of the age, gravidity and parity of pregnant women.  **Concept**  This review will consider studies that reported risk factors for APOs including demographic characteristics, social, ecological, clinical, behavioural etc. in RPMs. The eligibility of studies in this review will be based on satisfying two criteria: first, should report on the risk factors of APOs and two, these risk factors should be used in RPMs to predict APOs. APO in this review will be defined as all pregnancies resulting in undesirable/unwanted outcomes for the mother, newborn or both during labour, delivery and postpartum. Other terminologies for APOs will include adverse birth outcomes, poor birth outcomes, poor neonatal outcomes, poor maternal outcomes, poor obstetric outcomes, adverse obstetric outcomes, adverse maternal outcomes, adverse newborn outcomes and adverse neonatal outcomes.    **Context**  This review will consider studies conducted in LMICs based on the World Bank’s classification (23). |
| Information sources | 9 | The search strategy will aim to retrieve both published and unpublished articles. The following databases will be searched: PubMed/Medline, Cochran Library, Web of Science and Scopus.  This review will adopt a three-stage search strategy based on JBI guidelines (26): phase one (initial search), phase two (second search using identified keywords) and phase three (third search which includes a review of references identified articles for critical appraisal). In phase one, an initial search of databases was conducted to find articles on the topic. A comprehensive search technique for MEDLINE (PubMed) was created using the text words found in titles and abstracts as well as index keywords of relevant studies (Table 1). This will be followed up with an analysis of text words in the title, abstract and index terms adopted for the article. This will enhance the development of a search strategy that will suit each database source. In the second phase, the identified keywords and index terms will be used to search for literature in all relevant databases. In the third phase, a comprehensive search will be conducted by reviewing the reference list of all the identified articles for critical appraisal for additional eligible studies (snowballing). |
| Search strategy | 10 | The search strategy will aim to retrieve both published and unpublished articles. The following databases will be searched: PubMed/Medline, Cochran Library, Web of Science and Scopus.  This review will adopt a three-stage search strategy based on JBI guidelines (26): phase one (initial search), phase two (second search using identified keywords) and phase three (third search which includes a review of references identified articles for critical appraisal). In phase one, an initial search of databases was conducted to find articles on the topic. A comprehensive search technique for MEDLINE (PubMed) was created using the text words found in titles and abstracts as well as index keywords of relevant studies (Table 1). This will be followed up with an analysis of text words in the title, abstract and index terms adopted for the article. This will enhance the development of a search strategy that will suit each database source. In the second phase, the identified keywords and index terms will be used to search for literature in all relevant databases. In the third phase, a comprehensive search will be conducted by reviewing the reference list of all the identified articles for critical appraisal for additional eligible studies (snowballing). |
| Study records: |  |  |
| Data management | 11a | After the search, all identified studies will be collated and uploaded into Rayyan Software ([http://rayyan.qcri.org](http://rayyan.qcri.org/)) and all records of duplicates will be removed. |
| Selection process | 11b | The title and abstract of the studies screening will be done by two independent reviewers based on the inclusion criteria for the review as well as addressing the research questions. All potentially eligible studies which will be identified including those without a full text will be retrieved and their details will be imported into the JBI SUMARI software (25). The independent reviewers will retrieve the full text of all the selected studies and evaluate them based on the inclusion criteria. All studies that do not meet the inclusion criteria for the review will be excluded and reasons given as an appendix in the final scoping review report. All the studies that will be included will be critically reviewed independently by two reviewers and in instances where there is a disagreement between the two reviewers for the inclusion of a study, discussions will be made until a consensus is reached, and where necessary assessment of the full text will be done. If there is no agreement between the two reviewers, a third reviewer (senior colleague) will be invited for resolution. The PRISMA flow diagram (27) will be used for the presentation of the results of the search in the final scoping review. |
| Data collection process | 11c | Data extraction will be conducted by two independent reviewers using a standardized JBI SUMARI extraction tool. The tool will be used to train the research team members before it is used in the final work. The data that will be extracted will include the population (characteristics or predictors such as pregnant women, age, etc.) concept (risk prediction models), context (LMICs), study methods and designs, and other variables relevant to the review questions and objectives. The data extracted by the two independent reviewers will be compared and where there will be disagreements, it will be resolved through a discussion or a third reviewer will be invited to resolve it |
| Data items | 12 | List and define all variables for which data will be sought (such as PICO items, funding sources), any pre-planned data assumptions and simplifications |
| Outcomes and prioritization | 13 | List and define all outcomes for which data will be sought, including prioritization of main and additional outcomes, with rationale |
| Risk of bias in individual studies | 14 | This does not apply to a scoping review |
| Data synthesis | 15a | The extracted data from the studies will be presented using tables based on the objectives and the review questions. An initial summary of the characteristics of the studies including the study design, date of publication, population and authors’ geographical location will be described in the review. Quantitative data will be described descriptively and presented using frequencies. Tables will be used to describe adverse maternal and newborn outcomes, RPMs used to predict these adverse outcomes as well as the predictors that were used for the prediction. We will merge data from the same studies. |
|  | 15b |  |
|  | 15c | Not applicable |
|  | 15d | A narrative summary will be used to describe the results based on the research questions which guided the review which can guide future development, reporting and comparison of models and outcome selection of adverse maternal and newborn outcomes. We will publish the outcome of this scoping review in a peer-reviewed journal as well as present at either national or international conferences where possible. |
| Meta-bias(es) | 16 | Not applicable |
| Confidence in cumulative evidence | 17 | Not applicable |

*** It is strongly recommended that this checklist be read in conjunction with the PRISMA-P Explanation and Elaboration (cite when available) for important clarification on the items. Amendments to a review protocol should be tracked and dated. The copyright for PRISMA-P (including checklist) is held by the PRISMA-P Group and is distributed under a Creative Commons Attribution Licence 4.0.**

*From: Shamseer L, Moher D, Clarke M, Ghersi D, Liberati A, Petticrew M, Shekelle P, Stewart L, PRISMA-P Group. Preferred reporting items for systematic review and meta-analysis protocols (PRISMA-P) 2015: elaboration and explanation. BMJ. 2015 Jan 2;349(jan02 1):g7647.*
